# Potential for replacing warfarin with a direct oral anticoagulant for endoscopic mucosal resection in the colorectum: A multicenter, open‐label, randomized controlled trial

**DOI:** 10.1002/deo2.102

**Published:** 2022-02-21

**Authors:** Takuya Yamada, Toshio Kuwai, Yoshihiro Sasaki, Yuko Sakakibara, Toshio Uraoka, Motohiko Kato, Noriko Watanabe, Toshihisa Kimura, Akiko Kada, Akiko M. Saito, Naohiko Harada

**Affiliations:** ^1^ Department of Gastroenterology and Hepatology National Hospital Organization Osaka National Hospital Osaka Japan; ^2^ Department of Gastroenterology Osaka Rosai Hospital Osaka Japan; ^3^ Department of Gastroenterology National Hospital Organization Kure Medical Center and Chugoku Cancer Center Hiroshima Japan; ^4^ Department of Gastroenterology National Hospital Organization Disaster Medical Center Tokyo Japan; ^5^ Department of Gastroenterology National Hospital Organization Tokyo Medical Center Tokyo Japan; ^6^ Division of Gastroenterology and Hepatology, Department of Internal Medicine Keio University School of Medicine Tokyo Japan; ^7^ Department of Gastroenterology National Hospital Organization Mie Chuo Medical Center Mie Japan; ^8^ Department of Surgery National Hospital Organization Tsuruga Medical Center Fukui Japan; ^9^ Clinical Research Center National Hospital Organization Nagoya Medical Center Aichi Japan; ^10^ Department of Gastroenterology Clinical Research Institute, National Hospital Organization Kyushu Medical Center Fukuoka Japan

**Keywords:** direct oral anticoagulant, endoscopic mucosal resection, heparin bridging, randomized controlled trial, warfarin

## Abstract

**Objectives:**

This study aimed to evaluate the efficacy and safety of apixaban replacement (AR) as an alternative to heparin bridging (HB) in patients taking warfarin and scheduled for endoscopic mucosal resection (EMR) in the colorectum.

**Methods:**

This trial was conducted at seven institutes in Japan between May 2016 and May 2018. Enrolled patients had been taking oral warfarin and were diagnosed within 3 months with colorectal polyps for which EMR was indicated. Patients were randomly assigned to receive HB or AR. The primary endpoint was the incidence of postoperative bleeding. Secondary endpoints were the length of hospital stay, therapeutic endoscopy outcomes, and adverse events.

**Results:**

The planned sample size was 160 patients, but due to a decrease in the number of patients taking warfarin, the target number of cases could not be achieved within the case enrollment period, 44 cases were enrolled. They were divided into HB and AR groups. The incidence of postoperative bleeding was 15% (3/20) in HB and 0% in AR (P = 0.199). The total number of postoperative bleeding events was five in HB and none in AR. The length of hospital stay was significantly shorter in AR than in HB (median: 3.0 vs. 13.5 days, *p* < 0.001). There were no serious adverse events and no cerebral infarction/systemic embolism events.

**Conclusion:**

AR for colorectal EMR may prove safe and has the potential to shorten hospital stay and reduce medical costs, though we were unable to evaluate the primary endpoint due to insufficient sample size.

## INTRODUCTION

With the rapidly aging society in Japan and the recent Westernization of dietary habits, the number of patients receiving antithrombotic drugs is increasing. Similarly, the incidence of colorectal neoplasms, such as colorectal cancer and colorectal adenoma, is also increasing, and the number of patients undergoing colonoscopy is also increasing.^1^, ^2^, ^3^ Anticoagulants are the first choice for the prevention of cerebral infarction associated with atrial fibrillation, transient cerebral ischemic attack, and other cardiogenic cerebral embolisms. Warfarin and heparin have long been used for this purpose, but direct oral anticoagulants (DOACs) have been used since 2011.^2^, ^3^, ^4^, ^5^, ^6^ In the Japan Gastroenterological Endoscopy Society (JGES) guidelines,^7^ endoscopic mucosal resection (EMR) is indicated for colon polyps, and heparin substitution followed by withdrawal, referred to as heparin bridging (HB), is necessary for patients taking oral anticoagulants. However, HB has various limitations; the risk of postoperative bleeding is high, and it results in longer hospital stays. A high risk of postoperative bleeding after heparin replacement has been reported by the JGES in 2018.^8^ Several retrospective studies have evaluated the risk of HB,^9^, ^10^ but the precise effects of this strategy are not established.

Apixaban is not only superior to other DOACs in stroke prevention but also has a relatively low risk of gastrointestinal bleeding among DOACs.^6^, ^11^, ^12^, ^13^, ^14^, ^15^ In addition, since the time to reach the maximum plasma concentration is 3–3.5 h and the elimination half‐life is 6–8 h, it is not necessary to resume heparin, and the risk of postoperative bleeding is low. Apixaban substitution is an alternative treatment for patients with atrial fibrillation taking oral warfarin with a high risk of stroke; it can reduce postoperative bleeding and shorten the length of hospital stay following colorectal EMR. This study aimed to evaluate the efficacy and safety of apixaban replacement (AR) as an alternative to HB in patients taking warfarin and scheduled for EMR for the colorectal polyp.^16^

## METHODS

### Patient selection

Patients taking oral warfarin for non‐valvular atrial fibrillation or venous thrombosis with a colorectal polyp for which EMR was indicated, were recruited. The following inclusion criteria were applied: 1) scheduled to undergo EMR for a colorectal polyp (including hyperplastic polyps, adenoma, or early‐stage cancer), 2) taking oral warfarin for non‐valvular atrial fibrillation or venous thrombosis, with a prothrombin time‐international normalized ratio (PT‐INR) controlled at 3.0 or below, 3) aged 20 years or older at the time of informed consent, and 4) provided written consent of his or her own free will. The following exclusion criteria were applied: 1) hemorrhagic diathesis (platelet count ≤ 50,000), 2) receiving maintenance dialysis, 3) pregnant or potentially pregnant (women), 4) unable to reduce the antiplatelet regimen to a single drug, and 5) deemed unsuitable for participation by the principal investigator or other investigators.

### Study design

This was an open‐label, randomized, parallel‐group, controlled trial conducted at seven institutes of the National Hospital Organization.

The primary endpoint was the proportion of postoperative bleeding (where postoperative bleeding is defined as overt hematochezia within the observation period [4 weeks] after EMR). Secondary endpoints included the hospitalization period, therapeutic endoscopy outcomes (proportions of en‐bloc resection, perforation, and intraoperative bleeding), the incidence of cerebral infarction/systemic embolism events, the incidence of serious adverse events, and the proportion of postoperative therapeutic endoscopy.

This study was in compliance with the World Medical Association's Declaration of Helsinki, Ethical Guidelines for Medical and Health Research Involving Human Subjects, and Act on the Protection of Personal Information. It was approved by the National Hospital Organization Central Review Board for Clinical Trials (April 19, 2016). The study followed the CONSORT (Consolidated Standards of Reporting Trials) 2010 statement (Supporting Information 1)^17^ and was registered in the University Hospital Medical Information Network Clinical Trials Registry (UMIN000021947) accepted by the International Committee of Medical Journal Editors. All patients provided written informed consent to undergo the procedures and participate in this study.

### Study outline

**FIGURE 1:**
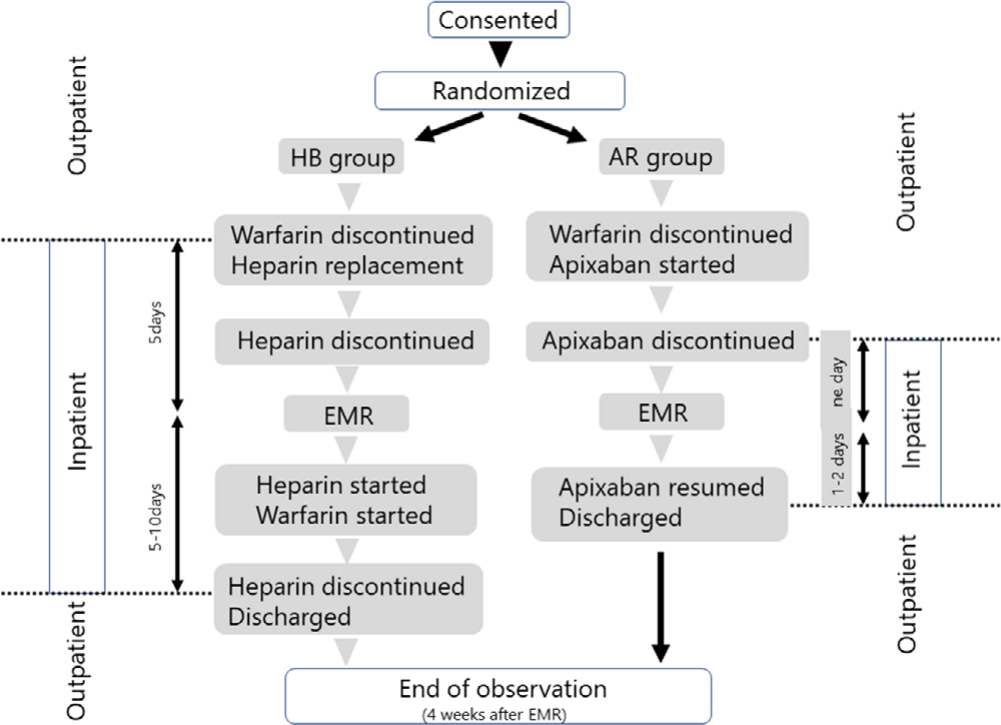
Design of this study. AR, apixaban replacement; EMR, endoscopic mucosal resection; HB, heparin bridging

Figure 1 provides an outline of this study. Patients—‐those who were taking oral warfarin for non‐valvular atrial fibrillation or venous thrombosis on an outpatient basis and diagnosed with a colorectal polyp (including adenoma or early‐stage cancer) for which EMR was indicated—‐ were assigned to one of two groups using an electronic data capture system by the minimization method. Adjustment factors were; hospital, use/non‐use of oral antiplatelet drugs. In the HB group, patients received warfarin according to the Guidelines for the management of anticoagulant and antiplatelet therapy in cardiovascular disease (JCS 2009).^18^ Warfarin was discontinued and heparin was initiated upon hospitalization 5 days before treatment. On the day of the operation, EMR was performed 6 h after the suspension of intravenous heparin administration. At 3 h after treatment, heparin administration was resumed. Oral warfarin was resumed at the preoperative dose on the next day after the absence of postoperative bleeding was confirmed. Oral antiplatelet drugs that were discontinued prior to EMR were resumed along with warfarin. PT‐INR was monitored, as appropriate; heparin administration was discontinued, and the patient was discharged after reaching the therapeutic range (2.0–3.0). Each heparin regimen was started at a dose of 10,000 units/day and calibrated to obtain a partial thromboplastin time of 2.0 times the baseline value. In the AR group, warfarin was discontinued 14 days before treatment, and apixaban was started when the PT‐INR dropped to ≦ 2.0. The standard apixaban dosage is 5.0 mg twice per day, but it is 2.5 mg twice per day for patients meeting at least two of the following criteria: age ≥ 80 years, body weight ≤ 60 kg, or serum creatinine ≥ 1.5 mg/dl. Patients were hospitalized the day before treatment, and apixaban was discontinued that evening. EMR was performed on the following day. Oral apixaban was resumed, and the patient was discharged after the absence of postoperative bleeding the morning after EMR was confirmed. Oral antiplatelet drugs that were discontinued prior to EMR were resumed at the same time as apixaban. Patients in both groups had an outpatient consultation 28 days after treatment to check for postoperative bleeding. In the event of positive findings, the hemorrhage site was verified by colonoscopy.

### Management of antiplatelet drugs

Any oral antiplatelet drugs were suspended 1 day before treatment (3 days before for aspirin or 5 days before for thienopyridine derivatives). However, patients who were unable to discontinue their antiplatelet regimen were allowed to proceed in cases of oral aspirin and/or cilostazol. Regimens consisting of multiple oral antiplatelet drugs were replaced with an aspirin or cilostazol regimen.

### EMR procedure

EMR was performed by standard procedures. However, the following provisions were strictly followed: a) no cold polypectomies and b) clipping after resection may be performed at the facility's discretion. Colonoscopy was performed when hematochezia was confirmed to identify the source of bleeding and achieve hemostasis, to the extent possible.

### Sample size determination

The hypothesis that the proportion of postoperative bleeding is lower in the AR group than in the HB group was evaluated. The target study population was 160 patients, with 80 in the standard treatment HB group (the control group) and 80 in the experimental AR group.

Briefly, the proportion of postoperative bleeding was expected to be 20% in the HB group, as prior studies have reported incidences of 10% to 23% after colon EMR with HB.^9^, ^10^ The proportion was predicted to be 5% in the experimental treatment group, based on the observation that none of the 10 patients who underwent EMR while taking DOACs at the National Hospital Organization Osaka National Hospital from 2012 to 2014 experienced postoperative bleeding. The estimated sample size was 76 per group for a one‐sided significance level of 0.025 and a statistical power of 0.80 to detect a beneficial effect in the experimental treatment group. In anticipation of dropouts, the sample size was set to *n* = 80/group (160 patients in total).

### Statistical analysis

In accordance with the intention‐to‐treat (ITT) principle, the ITT set consisted of all cases registered and allocated to groups in the present study. The full analysis set (FAS) consisted of all cases in the ITT set meeting the eligibility criteria for whom, at minimum, study treatment was started, and at least one observation was recorded after randomization. The safety analysis set consisted of all cases who began the treatment. Efficacy was primarily analyzed in the FAS.

The proportion of postoperative bleeding in each group was compared by the continuity‐adjusted chi‐square test. The hospitalization periods were compared using the Wilcoxon test. For EMR outcomes, the incidence of cerebral infarction/systemic embolism events and the proportion of postoperative therapeutic endoscopy, point estimates for differences, and 95% confidence intervals were calculated. Frequencies of adverse events and serious adverse events were evaluated in each group. As an additional analysis, the incidence of postoperative bleeding by the group was compared using the Poisson regression model with groups for fixed effect. The incidence of postoperative bleeding was recorded as the total number of events over the total observation days for each group. Significance testing was two‐sided, with a significance threshold of 0.05. All statistical analyses were conducted with SAS version 9.4 (SAS Institute, Cary, NC, USA).

## RESULTS

### Baseline clinical characteristics

**FIGURE 2:**
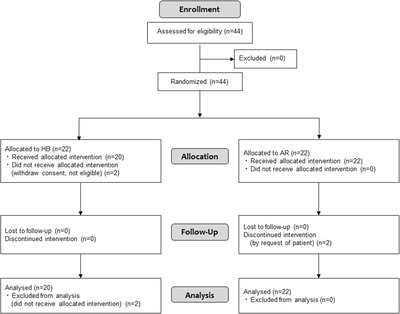
Flow chart of this study result. *Discontinued for the following reasons: two patients for withdrawal of consent. AR, apixaban replacement; HB, heparin bridging

We recruited 44 patients during the period from May 2015 to May 2018 at seven institutions; 22 patients were assigned to the HB group and 22 patients were assigned to the AR group. The target sample size was not reached and there may, therefore, be insufficient power to detect significant differences between groups. Two patients in the AR group did not receive the allocated intervention because they withdrew their consent and were not eligible; accordingly, analyses for FAS and safety included 22 cases in the HB group and 20 cases in the AR group (Figure 2). Table 1 summarizes the characteristics of patients at baseline. The male‐female ratios were 15:5 and 20:2, respectively, and the median ages were 75 and 76 years in the HB and AR groups. There were 11 cases (55%) in the HB group and 10 cases (45.5%) in the AR group with a CHADS2 score of ≥ 2 (indicating a risk of atrial fibrillation). In total, 47 polyps were resected in the HB group and 65 were resected in the AR group. Laboratory values (platelet count, PT‐INR, and creatinine clearance) did not show a big difference between the two groups. Table 2 shows the characteristics of resected lesions at baseline. The median polyp sizes were 5.0 and 6.0 mm, respectively, in the HB and AR groups. Histopathology and polyp locations did not show a big difference between the two groups.

**TABLE 1:** Baseline characteristics of the patients

	HB (*N* = 20)	AR (*N* = 22)
Age, median (IQR)	75.0 (66–84)	76.0 (58–93)
Gender (male/female)	15 /5	20 /2
CHADS_2_ score, median (IQR)	2 (0–3)	1 (0–5)
Antiplatelet use	4 (20.0％)	5 (22.7%)
Total number of polyps	47	65
Laboratory values mean ± SD		
Platelet count (10^4^μl)	17.8 ± 5.2	18.3 ± 5.3
PT‐INR	2.1 ± 0.49	2.0 ± 0.49
Creatinine clearance (ml/min)	54.4 ± 18.1	64.8 ± 29.8

Abbreviations: AR, apixaban replacement; HB, heparin bridging; PT‐INR, prothrombin time‐international normalized ratio.

**TABLE 2:** Baseline characteristics of the resected lesions

	HB (*N* = 47)	AR (*N* = 65)
Polyp size (mm), median (IQR)	5.0 (3–22)	6.0 (2–26)
Histopathology, *n* (%)		
Hyperplastic	1 (2.1)	2 (3.1)
Adenoma	44 (93.6)	56 (86.1)
Cancer	2 (4.3)	7 (10.8)
Location of polyps, *n* (%)		
Cecum	3 (6.4)	5 (7.7)
Ascending colon	10 (21.3)	10 (15.4)
Transverse colon	10 (21.3)	16 (24.6)
Descending colon	4 (8.5)	7 (10.8)
Sigmoid colon	15 (31.9)	16 (24.8)
Rectum	5 (10.6)	11 (16.9)

Abbreviations: AR, apixaban replacement; HB, heparin bridging.

### Study outcomes

The proportion of postoperative bleeding was 15% (3/20 cases) in the HB group, and no postoperative bleeding was observed in the AR group, and there was no significant difference (*p* = 0.199) (Table 3). In one case, postoperative bleeding occurred repeatedly. The total number of postoperative bleeding events was significantly higher in the HB group (*n* = 5) than in the AR group (*n* = 0; *p* < 0.001). The median hospital stay was 13.5 days in the HB group and 3 days in the AR group, and this difference was significant (*p* < 0.001). Intraoperative hemorrhage and intraoperative perforation during endoscopic treatment were not observed in either group. Neither cerebral infarction/systemic embolism nor serious adverse events were observed in either group. The postoperative bleeding cases are shown in Table 4. All cases were observed in the HB group. The platelet count and PT‐INR were normal, and no antiplatelet agents were used. Prophylactic clipping was performed in all cases, and the onset of postoperative bleeding in all cases was within 1 week after treatment.

**TABLE 3:** Comparison of efficacy and safety

	HB (*N* = 20)	AR (*N* = 22)	*p*‐value
Proportion of postoperative bleeding (%)	15% (3/20)	0% (0/22)	0.199*
Number of postoperative bleeding	5	0	<0.001^†^
Length of hospital stay (day) median (range)	13.5 (6–20)	3.0 (3–6)	<0.001^#^
Endoscopic treatment			
Perforation	0	0	NA
Intraoperative bleedings	0	0	NA
Arterial thromboenbolism	0	0	NA
Deep‐vein thrombosis	0	0	NA
Pulmonary embolism	0	0	NA
Major bleeding	0	0	NA
Minor bleeding	1	0	NA

Abbreviations: AR, apixaban replacement; HB, heparin bridging; NA, not applicable.

* :Continuity‐adjusted chi‐square test.

^†^ : The incidence of postoperative bleeding by the group was compared using the Poisson regression model with groups for fixed effect. The incidence of postoperative bleeding was the total number of events per total observation days for each group.

^#^ : Wilcoxon test.

**TABLE 4:** Cases of postoperative bleeding

					**Polyp characteristics**					
**Group**	**Gender**	**Age**	**Platelet (10^4^ μl)**	**PT‐INR**	**Size (mm)**	**Site**	**Onset of bleeding (POD)**	**Prophylactic clipping**	**Number of bleeding**	**Hospital stay (days)**
HB	F	84	13.8	2.68	22	C	1, 3, 7	Yes	3	19
HB	F	68	7.1	2.0	5	C	4	Yes	1	12
HB	F	64	11.5	1.71	5	S	2	Yes	1	13

Abbreviations: C, cecum; F, female; HB, heparin bridging; POD, post‐operation day; PT‐INR, prothrombin time‐international normalized ratio; S, sigmoid colon.

## DISCUSSION

We performed the first direct comparison of HB with DOACs replacement in colorectal EMR for patients receiving warfarin. In particular, we evaluated the effects and risks of apixaban in colorectal EMR during anticoagulant therapy.

HB is currently considered a standard approach for patients taking oral warfarin who require therapeutic endoscopy.^7^, ^19^, ^20^ ASGE guidelines and BSG‐ESGE guidelines recommend that HB be performed when colorectal EMR is performed for patients at high risk of thromboembolic events. However, this strategy is still limited by postoperative bleeding and long hospital stays.^9^, ^10^, ^21^ In addition, there are several reports on the risk of HB in Western countries.^22^, ^23^, ^24^

DOACs have been widely used since they were approved for stroke prevention in patients with non‐valvular atrial fibrillation^2^, ^5^, ^6^, ^11^ and the treatment of venous thromboembolism.^3^, ^4^ DOACs are characterized by a rapid onset and offset of action, an anticoagulant effect at certain doses, and a shortened half‐life. These features simplify the perioperative management of anticoagulant therapy, reduce the time between drug interruptions, and avoid the cost and inconvenience of HB.

López‐López et al.^6^ reported a network meta‐analysis of the stroke‐preventive effects of DOACs in patients with AF and showed that apixaban had a lower risk of stroke or systemic embolism than other DOACs, as well as a lower risk of major bleeding and gastrointestinal bleeding. Unlike other DOACs, dabigatran is a prodrug and is present in the gastrointestinal tract as a prodrug.^25^ It is possible that dabigatran is transformed into its active form by intestinal bacteria, thus inhibiting hemostasis. In addition, the above‐mentioned meta‐analysis reported that the risk of gastrointestinal bleeding is higher with dabigatran and edoxaban than with warfarin,^6^ so replacement with these two drugs may be avoided.

In this trial, the proportion of postoperative bleeding was 15% in the HB group and 0% in the AR group. In the HB group, cases of repeated postoperative bleeding were observed. There was no significant difference in overall postoperative bleeding events between groups owing to the small number of cases, but the lower frequency in the AR group suggests that AR may suppress postoperative bleeding. The median length of hospital stay was significantly lower in the AR group, which could reduce the mental and economic burden on patients. Regarding the safety of AR, no serious adverse events were observed in 20 patients, and EMR was performed in all patients. In addition, cerebral infarction and systemic embolism did not occur during follow‐up, suggesting that AR is safe.

In Japan, where the population is aging, rising medical expenses are a problem that needs to be solved. Polypectomy reduces medical costs by reducing morbidity and mortality associated with colorectal cancer.^26^, ^27^, ^28^ Also, reducing the cost of removing colon polyps is considered very important. The cost of medical care in Japan required for resection of colon polyps is about 450,000 Yen for HB and about 200,000 Yen for AR.

This study had some limitations. First, the target sample size of 160 cases was not reached; instead, 44 cases were enrolled. The number of eligible patients was lower than expected because warfarin was already replaced with DOACs at the start of the study in many cases, and the DOACs were limited to non‐valvular atrial fibrillation. Although indications for venous thrombosis were added during the study, the number of cases did not increase and this was stopped. Although there were no significant differences in the proportion of postoperative bleeding, the number of postoperative bleeding events was lower and the hospitalization stays were shorter in the AR group than in the HB group. Increases in medical costs, days of hospitalization, and medical costs have become major issues in Japan. These beneficial effects of AR suggest that this strategy could contribute to alleviating these general issues. Second, although we detected a significant difference in the number of bleeding events, this was an additional analysis. However, clinically, there are cases in which a single patient bleeds many times, and the physical and mental burden on both patients and medical professionals is high in these cases. Therefore, the potential for AR to reduce the number of bleeding events may be very useful. Third, although cerebrovascular diseases were not observed in either the AR or HB groups, statistical evaluation was difficult because of the short observation period of about 2 months. The replacement of warfarin with apixaban before endoscopic treatment has not been evaluated previously. Although the results of this study could not show the effectiveness of this strategy, they do support its safety.

In conclusion, the replacement of warfarin with apixaban for colorectal EMR may prove safe and has the potential to shorten hospital stay and reduce medical costs, though we were unable to evaluate the primary endpoint in this study due to insufficient sample size.

## CONFLICT OF INTEREST

Author Toshio Uraoka is a Deputy EIC of DEN Open. The rest of the authors do not have any conflict of interest.

## FUNDING INFORMATION

This study is being conducted using an operating expense grant for research from the National Hospital Organization.

## Supporting information


**Supporting Information 1**. CONSORT 2010 checklist
